# Near-Complete Genome Sequence of *Thalassospira* sp. Strain KO164 Isolated from a Lignin-Enriched Marine Sediment Microcosm

**DOI:** 10.1128/genomeA.01297-16

**Published:** 2016-11-23

**Authors:** Hannah L. Woo, Kaela B. O’Dell, Sagar Utturkar, Kathryn R. McBride, Marcel Huntemann, Alicia Clum, Manoj Pillay, Krishnaveni Palaniappan, Neha Varghese, Natalia Mikhailova, Dimitrios Stamatis, T. B. K. Reddy, Chew Yee Ngan, Chris Daum, Nicole Shapiro, Victor Markowitz, Natalia Ivanova, Nikos Kyrpides, Tanja Woyke, Steven D. Brown, Terry C. Hazen

**Affiliations:** a Department of Civil and Environmental Engineering, University of Tennessee, Knoxville, Tennessee, USA; b Department of Microbiology, University of Tennessee, Knoxville, Tennessee, USA; c Graduate School of Genome Science and Technology, University of Tennessee, Knoxville, Tennessee, USA; d DOE Joint Genome Institute, Walnut Creek, California, USA; e Biosciences Division, Oak Ridge National Laboratory, Oak Ridge, Tennessee, USA; f Department of Earth and Planetary Sciences, University of Tennessee, Knoxville, Tennessee, USA

## Abstract

*Thalassospira* sp. strain KO164 was isolated from eastern Mediterranean seawater and sediment laboratory microcosms enriched on insoluble organosolv lignin under oxic conditions. The near-complete genome sequence presented here will facilitate analyses into this deep-ocean bacterium’s ability to degrade recalcitrant organics such as lignin.

## GENOME ANNOUNCEMENT

Lignin derived from the plant cell wall is difficult for microbes to degrade due to its complex, aromatic, and heterogeneous structure. The physiochemical conditions and microbial controls on lignin degradation is relatively unknown. This knowledge gap is especially ill studied in marine environments, where lignin can enter as allochthonous organic matter through rivers (1). Few microorganisms other than white-rot fungi have been found to degrade lignin (2). In deep marine environments, where extreme oligotrophy and salinity can limit fungal growth, bacterial microorganisms may be primarily responsible for any lignin degradation. A better understanding of marine lignin degradation by microbes may discover potentially novel and stress-tolerant biocatalysts for lignin valorization application in industry.

Seawater and sediment were sampled from the Nile Deep Sea Fan, an oxic and hypersaline deep-ocean environment at 700-m depth (3). The sample was then enriched with 0.05% organosolv lignin for several weeks and then serially diluted and plated onto minimal media (ONR7a–DSMZ medium 950) agar containing 0.05% weight/volume alkali lignin. A colony appeared on the agar within two weeks of being incubated at 14°C that was later identified as belonging to the genus *Thalassospira* by 16S rRNA gene PCR and Sanger sequencing.

Genomic DNA was isolated from KO164 using a MoBio UltraClean extraction kit, and sequencing was performed at the Joint Genome Institute (JGI, Walnut Creek, CA, USA). A Pacific Biosciences standard template preparation protocol was used for creating >10-kb libraries. Genome sequencing was performed using a PacBio RS II instrument as described previously, using C4 chemistry and polymerase version P6 (4, 5). The sequencing run generated 166,402 filtered subreads totaling 718.9 Mb. All general aspects of library construction and sequencing performed at the JGI can be found at http://www.jgi.doe.gov. The raw reads were assembled using HGAP version: 2.3.0 (6). The final assembly contained two contigs, with a total genome size of 4.81 Mb. The input read coverage was 87.3×. Gene identification and annotation were performed at Oak Ridge National Laboratory using methods described previously (7).

The genome contains 4,472 candidate protein-coding genes and includes putative oxidoreductases, aromatic-ring opening dioxygenases, and peroxidases which may present targets for follow-up studies. Based on a BLASTn search of its four 16S rRNA gene copies, KO164 was most similar to *Thalassospira xiamenensis*, a strain isolated from oil-contaminated coastal water (8) and *T. permensis*, a halotolerant naphthalene-degrading strain from soil (9). In Biolog growth assays, KO164 was halotolerant up to 8% NaCl and grew optimally at pH 9.5. Among 200 different carbon sources tested, KO164 grew best in l-glutamate, acetic acid, l-pyroglutamate, malate, l-ornithine, glycyl-l-proline, itaconic acid, glycyl-l-glutamate, *N*-acetyl-l-glutamate, and l-glutamine. KO164 possesses the pathways to produce l-glutamate from alpha-ketoglutarate and ammonium but benefits from the exogenous addition of glutamate or glutamine. This genome sequence will facilitate studies in the breakdown of lignin and broader carbon and nitrogen cycling analyses of marine microbiomes. In addition, insights into growth in high-pH environments will be enabled by comparative genomics studies with other alkaliphiles such as *Caldalkalibacillus thermarum* strain TA2.A1 (10) and *Clostridium paradoxum* strain JW-YL-7 (11). 

### Accession number(s).

This whole-genome shotgun project has been deposited at DDBJ/ENA/GenBank under the accession number MAJC00000000.
